# Editorial: Early Genetic and Clinical Diagnosis in MEN1

**DOI:** 10.3389/fendo.2020.00218

**Published:** 2020-04-15

**Authors:** Delmar M. Lourenço, Wouter W. de Herder

**Affiliations:** ^1^Endocrine Genetics Unit (LIM-25), Endocrinology Division, Hospital das Clínicas, University of São Paulo School of Medicine, São Paulo, Brazil; ^2^Endocrine Oncology Division, Institute of Cancer of the State of São Paulo, São Paulo, Brazil; ^3^Sector Endocrinology, Department of Internal Medicine, ENETS Centre of Excellence, Erasmus MC Cancer Institute, Erasmus MC - University Medical Center, Rotterdam, Netherlands

**Keywords:** multiple endocrine neoplasia type 1, *MEN1* gene, early diagnosis, screening, young, adolescent

Multiple endocrine neoplasia type 1 (MEN1, OMIM 131100) is an autosomal dominant inherited syndrome caused by germline mutations in the *MEN1* gene that predisposes carriers to variable risk of development of tumors in diverse non-endocrine and endocrine organs. The main MEN1-related endocrine tumors affect the parathyroid and pituitary glands, pancreatic islets and duodenal endocrine cells. Most *MEN1* carriers develop main tumors between 5 and 80 years with at least one them initiating around 20–30 years (50–60%) and, invariably, 94% of MEN1 carriers have developed one or more disease manifestations at the age of 50 years. MEN1 is associated with decreased life expectancy, which in 75% of patients is caused by disorder-related morbidity/mortality (1). Different clinical protocols have been recommended to allow the early diagnosis and treatment of MEN1-related tumors in *MEN1* mutation carriers (1–7). Although the Clinical Practice Guidelines for MEN1 has recommended periodic vigilance initiating at 5, 8, and 10 years of age (1), respectively, for pituitary adenoma, primary hyperparathyroidism (HPT) and neuroendocrine pancreatic tumors, there are only a few papers emphasizing clinical features, management and treatment of children and adolescents with MEN1 (3, 8–13).

In the present issue of the *Frontiers in Endocrinology*, this topic was widely discussed by two independent research groups (Kamilaris and Stratakis; de Laat et al.). Kamilaris and Stratakis presented an updated and complete review on the genetics and molecular pathogenesis, including indications for performing the *MEN1* mutational analysis, diagnosis and treatment of the main MEN1-related tumors in young patients. These authors revisited a number of papers addressing the occurrence of these tumors at young ages, reinforcing the importance and the pivotal role of periodic biochemical and radiological surveillance of MEN1 carriers (Kamilaris and Stratakis).

The DutchMEN group critically debated the current clinical diagnosis of MEN1, emphasizing the different outcomes of true MEN1 cases harboring *MEN1* mutations and phenocopies. The latter group is defined as, (i) MEN1-like phenotypes harboring germline mutations in other genes (*AIP* or CDKIs genes), and (ii) mutation-negative cases secondary to sporadic and casual co-occurrence of two MEN1 tumors (de Laat et al.). Also, these authors suggested less strict strategies of vigilance for phenocopies. In addition, de Laat et al. reported that a scenario of late MEN1 diagnosis involving MEN1 index cases and family members is yet highly prevalent in developed countries. Importantly, both the low rate of clinical suspicion of MEN1 by clinicians and the delayed genetic testing of family members of MEN1 patients were associated with higher morbidity and mortality. The prompt recognition of suspected clinical features by clinicians, accessible genetic testing, centralization and monitoring of MEN1 care through national registries were useful in Netherlands to diagnose MEN1 earlier (de Laat et al.).

As mentioned, the study of MEN1 in children and adolescents is still limited. Recently, Marx and Lourenço debated diverse questions and controversies on HPT occurring in young *MEN1* carriers as parathyroid pathophysiology, the differential diagnosis of HPT with other hypercalcemic genetic disorders, the importance of periodic vigilance and early diagnosis, criteria to indicate early parathyroid surgery and different treatment strategies (Marx and Lourenço). This debate gains strength due to the complete penetrance of HPT in MEN1, the high occurrence of this tumor as the first clinical manifestation and the frequent presence of HPT in adolescent and young adult MEN1 patients.

Also, the clinical and surgical experience of the Italian group in the follow-up and surgery of youngers with MEN1-related HPT has been recently reported (Marini et al.). These authors described 19 young MEN1 cases diagnosed with HPT before 20y, the surgical strategies adopted and discussed critically the risks and benefits of early parathyroidectomy (PTX) in MEN1, since the ideal timing to indicate PTX in such cases is still not known (Marini et al.). The same group, in a retrospective study, emphasized the efficacy reached by the total PTX (TPTX) approach followed by parathyroid auto-transplantation in 28 out of 38 HPT/MEN1 patients diagnosed before the age of 30 y, in comparison with nine other similar patients submitted to subtotal parathyroidectomy (SPTX) (Tonelli et al.). In the long-term follow up (11.8 ± 6.6 y), relatively low rates of recurrence (14 vs. 22%) were observed when patients were submitted, respectively, to either TPTX and SPTX, whereas the rate of permanent hypoparathyroidism was 17.9 vs. 22%, respectively (Tonelli et al.). In addition, early demineralization was documented in two young MEN1 cases with HPT, followed by recovery of bone mineral density (BMD) after PTX (Tonelli et al.). These data reinforced previous findings documenting both the early bone loss in young HPT/MEN1 cases and the positive impact of PTX on bone health, leading to a significant BMD increase 12 months after surgery (14–19).

Recently, a protocol applied to HPT/MEN1 patients followed up by the MD Anderson Cancer Center was reported including preoperative evaluation, intraoperative decision-making as to initial cases and reoperations, and the step by step SPTX procedures, elected as the preferential surgical approach (Nobecourt et al.). In 2019, a large Brazilian cohort of 161 HPT/MEN1 patients submitted to PTX between 1987 and 2018 was reported (Montenegro et al.). Among them, 94 patients (58.4%) were operated on between 2011 and 2018 including seven out of nine patients younger than 21 y (Montenegro et al.). These findings might suggest a tendency to a recent increase in the number of HPT/MEN1 adolescents submitted to PTX and they are most probably due to more accessible genetic testing and active periodic vigilance of *MEN1* carriers, contrasting with previous periods (20–22). Furthermore, based on four adolescent HPT/MEN1 cases that underwent unintentional less than SPTX, Montenegro et al. debated on the potential risks and benefits of less extensive PTX procedures applied to the specific subset of young HPT/MEN1 patients presenting a documented localized disease. The need for more extensive studies on this specific topic was stressed (Montenegro et al.).

In fact, some groups have reported experiences with less extensive parathyroid surgeries in MEN1, independent of age (23–27). These protocols might potentially minimize the risks of hypoparathyroidism in younger MEN1 patients in which peak bone mass was not yet reached. In contrast, less extensive PTX procedures would increase the risks of surgical failure, which may lead to persistent or early recurrent HPT. However, classical surgical protocols as mostly SPTX, and also TPTX, remain elective surgeries for MEN1 patients whereas more studies are necessary to validate the efficacy of less extensive surgeries in young MEN1 cases with HPT (Tonelli et al.; Nobecourt et al.; Montenegro et al.) (28–36).

Recently, Dantas et al. described a young MEN1 patient presenting hydrocephalus and intracranial hypertension caused by a giant prolactinoma as first clinical manifestations of MEN1. The importance of refining and reaching a more effective periodic screening program to avoid acute life-threatening events was mentioned. Also, an extensive review on giant prolactinoma occurring as part of MEN1 was offered (Dantas et al.).

Conversely, two research groups from Italy and Brazil collaborated in this topic in *Frontiers in Endocrinology* amplifying the strict spectrum of original studies on MEN1-related tumors occurring at young ages suggesting potential peculiarities of the management and treatment of such cases (Marini et al.; Tonelli et al.; Montenegro et al.; Dantas et al.). However, due to the rarity of this disorder and the few studies focusing on a large number of affected young *MEN1* carriers, is not surprising the adoption of divergent protocols of management and treatment by different expert groups. These data indicate that more studies should be warranted to optimize strategies of follow-up, attempting to reach more secure and effective decision-making concerning treatment of the young patients with MEN1 syndrome.

Thus, overall, the papers of Kamilaris and Stratakis, de Laat et al., Marx and Lourenço, Nobecourt et al. updated, commented and transmitted important experiences attempting to amplify our knowledge on the present Research Topic in *Frontiers in Endocrinology*.

## Author Contributions

DL concepted, designed, and wrote the editorial. WH collaborated with improvements to the editorial writing.

### Conflict of Interest

The authors declare that the research was conducted in the absence of any commercial or financial relationships that could be construed as a potential conflict of interest.
